# Type B Chloramphenicol Acetyltransferases Are Responsible for Chloramphenicol Resistance in *Riemerella anatipestifer*, China

**DOI:** 10.3389/fmicb.2017.00297

**Published:** 2017-03-01

**Authors:** Li Huang, Hui Yuan, Ma-Feng Liu, Xin-Xin Zhao, Ming-Shu Wang, Ren-Yong Jia, Shun Chen, Kun-Feng Sun, Qiao Yang, Ying Wu, Xiao-Yue Chen, An-Chun Cheng, De-Kang Zhu

**Affiliations:** ^1^Research Center of Avian Diseases, College of Veterinary Medicine of Sichuan Agricultural UniversityChengdu, China; ^2^Key Laboratory of Animal Disease and Human Health of Sichuan ProvinceChengdu, China; ^3^Institute of Preventive Veterinary Medicine, Sichuan Agricultural UniversityChengdu, China

**Keywords:** *Riemerella anatipestifer*, chloramphenicol acetyltransferase, antibiotics resistance, homologous recombination, site-directed mutagenesis

## Abstract

*Riemerella anatipestifer* causes serositis and septicaemia in domestic ducks, geese, and turkeys. Traditionally, the antibiotics were used to treat this disease. Currently, our understanding of *R. anatipestifer* susceptibility to chloramphenicol and the underlying resistance mechanism is limited. In this study, the *cat* gene was identified in 69/192 (36%) *R. anatipestifer* isolated from different regions in China, including *R. anatipestifer* CH-2 that has been sequenced in previous study. Sequence analysis suggested that there are two copies of *cat* gene in this strain. Only both two copies of the *cat* mutant strain showed a significant decrease in resistance to chloramphenicol, exhibiting 4 μg/ml in the minimum inhibitory concentration for this antibiotic, but not for the single *cat* gene deletion strains. Functional analysis of the *cat* gene via expression in *Escherichia coli* BL21 (DE3) cells and *in vitro* site-directed mutagenesis indicated that His79 is the main catalytic residue of CAT in *R. anatipestifer*. These results suggested that chloramphenicol resistance of *R. anatipestifer* CH-2 is mediated by the *cat* genes. Finally, homology analysis of types A and B CATs indicate that *R. anatipestifer* comprises type B3 CATs.

## Introduction

*Riemerella anatipestifer* is a gram-negative, non-flagellar bacterium belonging to the *Flavobacteriaceae* family of bacteroidetes that causes serositis and septicaemia in domestic ducks, geese, and turkeys. Currently, the fatality rate of *R. anatipestifer*-infected ducks has reached up to 75%, thereby resulting in significant economic losses in the duck industry (Ruiz and Sandhu, 2013).

The extensive use of antibiotics for the treatment and prevention of serositis and septicaemia has resulted in multi-drug resistance in *R. anatipestifer* (Zhong et al., 2009). It was found that 40.5% of *R. anatipestifer* strains were resistant to chloramphenicol (Chen et al., 2010). Based on the reported whole genome sequence of *R. anatipestifer* (GenBank accession number: CP004020) (Wang et al., 2014), we searched for resistance genes in *R. anatipestifer* CH-2 in the Comprehensive Antibiotic Resistance Database (Aakra et al., 2010). We have identified two copies of the *cat* gene in *R. anatipestifer* CH-2, namely, G148_1769 and G148_1772.

The *cat* gene encodes chloramphenicol acetyltransferases (CATs) that inactivate the drugs choramphenicol, thiamphenicol, and azidamfenicol by acetylation, which is the most common mechanism conferring chloramphenicol resistance in bacteria (Schwarz et al., 2004). However, CAT did not inactivate florfenicol because of the replacement of the hydroxyl group at C3 by a fluor residue, and the acceptor site of the acetyl groups was structurally altered in florfenicol (Schwarz et al., 2004). In addition to acetylation inactivation of chloramphenicol, other enzymatic inactivation mechanisms, such as *O*-phosphorylated (Mosher et al., 1995) and hydrolysis reaction have been identified (Mosher et al., 1990; Tao et al., 2012). Moreover, resistance to chloramphenicol may also be due to mutations/modifications of the target site (Montero et al., 2007), decreased outer membrane permeability (Burns et al., 1989), and the presence of efflux pumps that often act as multidrug extrusion transporters (Daniels and Ramos, 2009), thereby reducing the effective intracellular drug concentration.

In this study, the *cat* gene was identified in 69/192 (36%) *R. anatipestifer* isolated from different regions in China by PCR. In order to verify whether *cat* gene was responsible for chloramphenicol resistance in *R. anatipestifer*, we constructed the *cat* gene deletion strains, complement strains and assessed the protein enzyme activity.

## Materials and methods

### Bacterial strains, plasmids, and growth conditions

The bacterial strains and plasmids used in this study are listed in Table 1. *R. anatipestifer* strains were grown at 37°C in tryptic soybean broth (TSB, Oxoid) or tryptic soy agar (TSA, Oxoid) in an atmosphere of 5% CO_2_. *Escherichia coli* (*E. coli*) strains were grown on Luria-Bertani (LB, Oxoid) broth or agar at 37°C. When required, antibiotics were added at the following final concentrations (μg/ml): Chloramphenicol (Cm, Sigma), 25; cefoxitin (Cfx, Sigma), 1; kanamycin (Kan, Sigma), 100; ampicillin (Amp, Sigma), 100 or spectinomycin (Spc, Sigma), 70. Diaminopimelic acid (DAP, 50 μg/ml) to *E. coli* X7213λpir cultures (Edwards et al., 1998).

**Table 1:** **Strains and plasmids used in this study**.

**Strains**	**Description**	**Source or reference**
*Riemerella anatipestifer* ATCC 11845	Serotype 6	ATCC
*R. anatipestifer* CH-2	Serotype 2	Laboratory collection
RA-CH2Δ1769	RA-CH2Δ1769, Spc^R^	This study
RA-CH2Δ1772	RA-CH2Δ1772, Cfx^R^	This study
RA-CH2Δ1769Δ1772	RA-CH2Δ1769Δ1772, Spc^R^ Cfx^R^	This study
RA-ATCC11845 (pLMF02)	*R. anatipestifer* ATCC11845 carrying pLMF02, Amp^R^ Cfx^R^	This study
RA-ATCC11845 (pLMF02:: *cat*)	*R. anatipestifer* ATCC11845 carrying pLMF02:: 1769, Amp^R^ Cfx^R^	This study
RA-ATCC11845 (pLMF02:: *cat*^*H*79*A*^)	*R. anatipestifer* ATCC11845 carrying pLMF02:: H79A, Amp^R^ Cfx^R^	This study
***Escherichia coli*** **strains**	**Description**	**Source or reference**
X7232	*endA1 hsdR17 ($r_{K}^{-}$$m_{K}^{+}$) glnV44 thi-1 recA1 gyrA relA1Δ(lacZYA-argF)U169λpir deoR (Φ80dlac Δ(lacZ)M15)*	Roland et al., 1999
X7232 (pRE112:: 1769USD)	*E. coli* X7232 pRE112:: 1769USD, Spc^R^ Cm^R^	This study
X7232 (pRE112:: 1772UCD)	*E. coli* X7232 pRE112:: 1772UCD,Cfx^R^ Cm^R^	This study
X7213	*thi-1 thr-1 leuB6 glnV44 fhuA21 lacY1 recA1 RP4-2-Tc::Mu λpirΔasdA4Δzhf-2:: Tn10*	Roland et al., 1999
X7213 (pRE112:: 1769USD)	*E. coli* X7213 pRE112::1769USD, DAP, Spc^R^ Cm^R^	This study
X7213 (pRE112:: 1772UCD)	*E. coli* X7213pRE112:: 1772UCD, DAP Cfx^R^ Cm^R^	This study
BL21(DE3)	*E. coli* BL21(DE3), expressing host cell	Laboratory collection
BL21(DE3) (pET30a)	*E. coli* BL21(DE3) carrying pET30a, Kan^R^	This study
BL21(DE3) (pET30a:: *cat*-s)	*E. coli* BL21(DE3) carrying pET30a:: *cat*-s, Kan^R^	This study
BL21(DE3) (pET30a:: *cat*^*H*79*A*^-s)	*E. coli* BL21(DE3) carrying pET30a:: *cat*^*H*79*A*^-s, Kan^R^	This study
S17-1	*Thi-1 thr leu tonA lac Y supE recA*::RP4-2-Tc::Mu Kan^R^	Miller and Mekalanos, 1988
S17-1 (pLMF02)	S17-1 carrying pLMF02, Amp^R^ Cfx^R^	This study
S17-1 (pLMF02:: *cat*)	S17-1 carrying pLMF02:: *cat*, Amp^R^ Cfx^R^	This study
S17-1 (pLMF02:: *cat*^*H*79*A*^)	S17-1 carrying pLMF02:: *cat*^*H*79*A*^, Amp^R^ Cfx^R^	This study
**Plasmids**	**Description**	**Source or reference**
pET30a	pBR322 lacZ, IPTG-inducible promoter, Kan^R^	Laboratory collection
pET30a:: *cat*-s	pET30a carrying *cat* adding his tag from *R. anatipestifer* CH-2, Kan^R^	This study
pET30a:: *cat*^*H*79*A*^-s	pET30a carrying *cat*^H79A^ adding his tag from *R. anatipestifer* CH-2, Kan^R^	This study
pLMF02	shuttle vector transferred between *E. coli* and *R. anatipestifer* Amp^R^, Kan^R^	Liu et al., 2016
pLMF02:: *cat*	pLMF02 carrying *cat* from *R. anatipestifer* CH-2, Amp^R^ Cfx^R^	This study
pLMF02:: *cat*^*H*79*A*^	pLMF02 carrying *cat*^*H*79*A*^, Amp^R^ Cfx^R^	This study
pYES1new	YAC-BAC shuttle plasmid with Spc^R^	Laboratory collection
pRE112	sacB mobRP4 R6K ori Cm^R^,pRE112-T-vector	Laboratory collection
pRE112:: 1769USD	pRE112 carrying 1769USD from *R. anatipestifer* CH-2and plasmid pYES1new, Spc^R^ Cm^R^	This study
pRE112:: 1772UCD	pRE112 carrying 1772UCD from *R. anatipestifer* CH-2 and plasmid pCP29, Cfx^R^ Cm^R^	This study

*ATCC: American Type Culture Collection*.

### Detection of the *cat* gene in *R. anatipestifer* isolates

For this study, 192 *R. anatipestifer* isolates were collected from different regions of China. All isolates were identified using the Biolog Microbial Identification System (Biolog, Hayward, CA, USA), as well as PCR and biochemical analyses (data not shown). After lysing the bacteria in lysis buffer (0.5% NP-40, Sigma; 200 ng/ml proteinase K, Takara Biotechnology Co., Ltd. Dalian, China), the presence of the *cat* gene was determined by PCR analysis using primers cat-F1 and cat-R1 (Table 2).

**Table 2:** **Primers used in this study**.

**Primers**	**Description**	**Source and reference**
1769up-F	5′-ATTCCAGTTTTTCAAATTCAATTCTTCCCTA-3′	This study
1769up-R	5′-CTGTCCTGGCTGGTATTTAACATATTTAATTTACA-3′	This study
Spc-F	5′-ATATGTTAAATACCAGCCAGGACAGAAATGCC-3′	This study
Spc-R	5′-CTTCTTTTTATTATTTGCCGACTACCTTGGTGA-3′	This study
1769down-F	5′-CGGCAAATAATAAAAAGAAGGTTCCGAAAT-3′	This study
1769down-R	5′-TTGATGTGGCATTTGCCTGCAGAT-3′	This study
1769Ident-F	5′-TTTGCGAAGAAGCGGGCTAA-3′	This study
1769Ident-R	5′-CAAAGAGTTCCTCCGCCGCT-3′	This study
1769Big-F	5′-TACTTAACCCGCCATTTTGCCA-3′	This study
1769Big-R	5′-AACGGTAGCAACCCAAGCAGTG-3′	This study
1772up-F	5′-ATTCTCCAGAGTCGGATTCTGTTGAATTTTTTA-3′	This study
1772up-R	5′-GCTTCGGGGTCATTATATATTTAACATATTTAATTTACAAT-3′	This study
Cfx-F	5′-ATATGTTAAATATATAATGACCCCGAAGCAGGGT-3′	This study
Cfx-R	5′-GAACCTTCTTTTTATTAAGATTTTACTGAAGTTTGCATT-3′	This study
1772down-F	5′-TTCAGTAAAATCTTAATAAAAAGAAGGTTCCGAAATTC-3′	This study
1772down-R	5′-TCTAATAAACGATTTTTGGTGGGACACAACTTAC-3′	This study
1772Ident-F	5′-ATTTTGACGGATTTATTAGTTGTT-3′	This study
1772Ident-R	5′-TTCCGTATAAGCTATCTGAAAACT-3′	This study
1772Big-F	5′-AATTTTGAACTTAACCCGCC-3′	This study
1772Big-R	5′-ACTACGTCGTACAACATCGTATTG-3′	This study
16SrRNA-F	5′-CGAAAGTGATAAGTTAGCCACCT-3′	This study
16SrRNA-R	5′-GCAGCACCTTGAAAATTGTCC-3′	This study
cat-F1/ MF1	5′-GGGAATTCCATATGAAAAATTTCTTCGAAAGTC-3′	This study
cat-R1/MR2	5'-CCGCTCGAGTCAGTGGTGGTGGTGGTGGTGTTTCATTTTTCTAAAAAACTT-3′	This study
MR1	5′-ATATTTAGCACCTTGATTACCTG -3′	This study
MF2	5′-GTAATCAAGGTGCTAAATATGATT-3′	This study
cat-F2	5′-CATGCCATGGATGAAAAATTTCTTCGAAAGTC-3′	This study
cat-R2	5′-CCGCTCGAGTCATTTCATTTTTCTAAAAAACTT-3′	This study

### Construction of *R. anatipestifer* CH-2 *cat* deletion mutants and generation of *cat*^*H*79*A*^ mutant

The *cat* genes were deleted by homologous recombination using a suicide vector pRE112 (Kong et al., 2011) as described previously (Luo et al., 2015). Briefly, the right flanking sequence (~620 bp) and the left flanking sequence (~620 bp) of the target genes G148_1769 and G148_1772 were amplified using primers 1769up-F and 1769up-R, 1769down-F and 1769down-R, 1772up-F and 1772up-R, and 1772down-F and 1772down-R, respectively (Table 2). The 1,145 bp SpcR cassette and the 1,192 bp CfxR cassette were amplified from plasmid pYES1 (Luo et al., 2015) and pLMF01 (Liu et al., 2016) using primers Spc-F, Spc-R and Cfx-F, Cfx-R, respectively (Table 2). The SpcR cassette and the CfxR cassette were used for deletion of G148_1769 and G148_1772, respectively. The PCR fragments were overlapped using the PCR method (Xiong et al., 2006). The fused PCR fragments were ligated to suicide plasmid pRE112, respectively, to produce pRE112:: 1769USD (SpcR) and pRE112:: 1772UCD (CfxR). Subsequently, the recombinant plasmids were introduced into *R. anatipestifer* CH-2 by conjugation as described previously (Liao et al., 2015). The transconjugants were selected on TSA plates supplemented with Spc (40 μg/ml) or Cfx (1 μg/ml). The gene-deletion mutant strains, which were designated as RA-CH2Δ1769, RA-CH2Δ1772, and RA-CH2Δ1769Δ1772, were identified by PCR analysis.

The *cat*^*H*79*A*^ mutant was constructed by *in vitro* site-directed mutagenesis. The upstream and downstream mutated regions of the *cat* gene of *R. anatipestifer* CH-2 amplified using primers MF1, MR1 and MF2, MR2 his, respectively (Table 2). The fragments were fused by overlap extension PCR to yield the mutant gene *cat*^*H*79*A*^.

### Construction of the recombinant vector for complementation and expression

Complete *cat* and *cat*^*H*79*A*^ genes were amplified by PCR from *R. anatipestifer* CH-2 chromosomal DNA and by *in vitro* site-directed mutagenesis using primers catF2 and catR2, catF2, MR1 and MF2, catR2 (Table 2), for complementation. Complete *cat* and *cat*^*H*79*A*^ genes were amplified by PCR from *R. anatipestifer* CH-2 chromosomal DNA and by *in vitro* site-directed mutagenesis using primers MF1 and MR2 his, MF1, MR1 and MF2, MR2 his, respectively (Table 2), for expression of CAT and CAT^*H*79*A*^ proteins. The complementation fragments were purified and digested with *Nco*I and *Xho*I, and ligated to the pLMF02 plasmid digested with *Nco*I and *Xho*I. The expression fragments were purified and digested with *Nde*I and *Xho*I, and ligated with the pET30a plasmid digested with corresponding restriction endonucleases. The ligation mixtures were introduced into CaCl_2_-competent DH5α cells. Transformants were screened by PCR, and positive clones were sequenced.

### Construction of *R. anatipestifer* ATCC 11845 *cat* and *cat*^*H*79*A*^ complementary strains

The plasmids, pLMF02, pLMF02:: *cat*, and pLMF02:: *cat*^*H*79*A*^, were introduced into *R. anatipestifer* ATCC 11845, respectively, by the method described previously (Liao et al., 2015). The transconjugants were selected on TSA plates supplemented with Cfx (1 μg/ml) and Kan (40 μg/ml). The complementation strains, RA-ATCC11845 (pLMF02), RA-ATCC11845 (pLMF02:: *cat*), and RA-ATCC11845 (pLMF02:: *cat*^*H*79*A*^), were identified by PCR analysis.

### Expression and purification of CAT and CAT^H79A^ his-tagged proteins

Strains *E. coli* BL21 (DE3) (pET30a:: *cat*-s) and *E. coli* BL21 (DE3) (pET30a:: *cat*^*H*79*A*^ -s) were grown overnight in LB medium containing Kan (100 μg/ml). Stationary-phase cultures were diluted to an OD_600_ of 0.05 in 500 ml of LB medium containing Kan (100 μg/ml) and incubated with shaking at 37°C until the culture density reached an OD_600_ of 0.6. Cells were then induced with 0.4 mM of isopropyl β-D-1-thiogalactopyranoside (IPTG) and reincubated at 37°C. The cells were harvested by centrifugation for 10 min at 8,000 rpm at 4°C, and then the pellet was resuspended in lysis buffer (20 mM Tris-HCl, pH 8.0; 50 ml) and sonicated. The cell lysate was clarified by centrifugation to eliminate cell debris and then applied to a metal affinity resin column that was equilibrated with the same buffer. The column was successively washed with buffers containing 20 mM, 50 mM imidazole, and phosphate buffer (pH 4.4 and 5.0, respectively). Recombinant proteins were ultrafiltered with storage buffer (20 mM Tris-HCl, pH 7.8). The protein purity was assessed using sodium dodecyl sulfate polyacrylamide gel electrophoresis (SDS-PAGE) followed by Coomassie Blue staining. Protein concentration was determined using the BCA method with bovine serum albumin as the standard.

### Minimum inhibitory concentration (MIC) testing

Chloramphenicol MIC tests for deletion mutants and complementary strains were performed in 96-well microtiter plates according to the Clinical and Laboratory Standard Institute criteria (CLSI, 2015). *E. coli* ATCC 25922 was used as a quality-control strain. The turbidity of the inocula was adjusted to 10^7^ CFU/ml (100 μl/well). An inoculated broth containing no antibiotics was included as positive control, and a tube of uninoculated broth was used as negative control. The experiments were repeated three times.

### Determination of mRNA levels of the *cat* gene by real-time PCR (RT-PCR) analysis

To assess whether the *cat* gene of *R. anatipestifer* was regulated by chloramphenicol, the wild-type strain was grown with TSB with or without 1 μg/ml of chloramphenicol. Total RNA was isolated from strains grown to log phase (OD_600_ ≈ 0.8–1.0) by using the RNAiso Plus kit (TaKaRa). DNA was removed using RNase-Free DNase. cDNA was generated by using the Sensiscript RT kit (TaKaRa), according to the manufacturer's instructions. Real-time quantitative PCR (qPCR) was performed to measure *cat* mRNA levels using SYBR *Premix EX Taq* II (TaKaRa). The primers used in real-time qPCR analysis are listed in Table 2. The expression level of the *cat* gene was normalized to that of the *rec*A gene, which was used as reference. All PCR reactions were performed in triplicate. The efficiency of primer binding was determined by linear regression by plotting the cycle threshold (CT) value vs. the log of the cDNA dilution. Relative quantification of the transcript was determined using the comparative CT method (2^−ΔΔCT^), calibrated to *rec*A. The experiments were performed multiple times independently and generated comparable results. The findings are presented as fold-change relative to the mRNA expression levels of the control strains.

### CAT activity assay

CAT catalyzes the transfer of an acetyl group from acetyl-CoA to Cm, producing acetylated Cm and CoASH. The CATase activity was assayed based on the disappearance of acetyl-CoA during Cm acetylation (Kobayashi et al., 2015). The reaction mixture contained 0.25 ml of 0.2 M Tris-HCl (pH 7.8), 0.05 ml of 1 mM acetyl-CoA, 0.05 ml of 1 mM Cm, 0.05 ml of 10 mM DTNB [5,5′-dithio-bis (2-nitrobenzoic acid)], and 0.1 ml enzyme extract. The reaction was initiated by the addition of Cm. An increase in absorbance at a wavelength of 412 nm, which arises from 5-thio-2-nitrobenzoic acid, was derived from the reaction between free CoASH and DTNB. The concentration of 5-thio-2-nitrobenzoic acid was determined using its molar extinction coefficient at 412 nm (13,600 M^−1^ cm^−1^). The value was then used in the determination of the amount of CoASH produced during the reaction. One unit of enzyme activity is defined as the amount of activity catalyzing 1 μmol of acetyl transfer per min under the assay conditions.

### Softwares

The changes of mRNAs were expressed as fold expression and calculated using the comparative CT (2^−ΔΔCT^) method. The results of RT-PCR were performed using GraphPad Prism 6.0 software for Windows (GraphPad Software Inc., La Jolla, USA). Homology analysis of types A and B CATs based on amino acid identity using DNAMAN 8.0 (Lynnon-Biosoft, Ontario, Canada).

## Results

### Identification and sequence analysis of the *cat* gene in *R. anatipestifer* isolates

The *cat* gene was identified in 69/192 (36%) *R. anatipestifer* isolates collected from China, thereby suggesting that the *cat* gene was widely distributed among *R. anatipestifer* strains. Sequence analysis found that there are two copies of the *cat* gene (G148_1769: 1854900…1855529 and G148_1772: 1858427…1859056) in *R. anatipestifer* CH-2. There is no similarity between the *cat* gene from *R. anatipestifer* CH-2 and type A *cat* genes previously reported. However, the *cat* genes share 99–100% identity in *R. anatipestifer* strains reported in NCBI.

### MIC of chloramphenicol for *R. anatipestifer* CH-2 and other strains

To verify whether the *cat* genes of *R. anatipestifer* CH-2 were responsible for chloramphenicol resistance, the deletion strains and complementation strains were constructed. Table 3 showed that the chloramphenicol MICs of *R. anatipestifer* CH-2 and RA-CH2Δ1769 were 32 and 64 μg/ml, respectively. Compared to the MIC of the wild-type strain, the MIC of RA-CH2Δ1769 increased (Table 3). Similarly, another signal *cat* gene deletion strain RA-CH2Δ1772 had no obviously decreased in resistance to chloramphenicol, exhibiting 32 μg/ml in the minimum inhibitory concentration for chloramphenicol (Table 3). Thus, we supposed that the two copies of *cat* gene in *R. anatipestifer* CH-2 were involved in chloramphenicol resistance. The two copies of the *cat* gene deletion strain RA-CH2Δ1769Δ1772 was constructed. The level of chloramphenicol resistance was determined to be significantly reduced, 4 μg/ml.

**Table 3:** **Minimal inhibitory concentration (MIC) of chloramphenicol on ***R. anatipestifer*** and other strains**.

**Strain**	**MIC (μg/ml)**
RA-CH-2	32
RA-CH2Δ1769	64
RA-CH2Δ1772	32
RA-CH2Δ1769Δ1772	4
RA-ATCC11845(pLMF02)	<2
RA-ATCC11845(pLMF02:: *cat*)	32
RA-ATCC11845(pLMF02:: *cat*^*H*79*A*^)	<2

To further verify that the *cat* genes are related to chloramphenicol resistance in *R. anatipestifer*, shuttle plasmid pLMF02 with the *cat* gene was introduced into *R. anatipestifer* ATCC 11845, which is sensitive to chloramphenicol. It was restored the level of chloramphenicol resistance (Table 3). These results strongly suggested that the *cat* gene was responsible for chloramphenicol resistance in *R. anatipestifer*.

### The transcription of *cat* gene was increased in RA-CH2Δ1769

**Figure 1:**
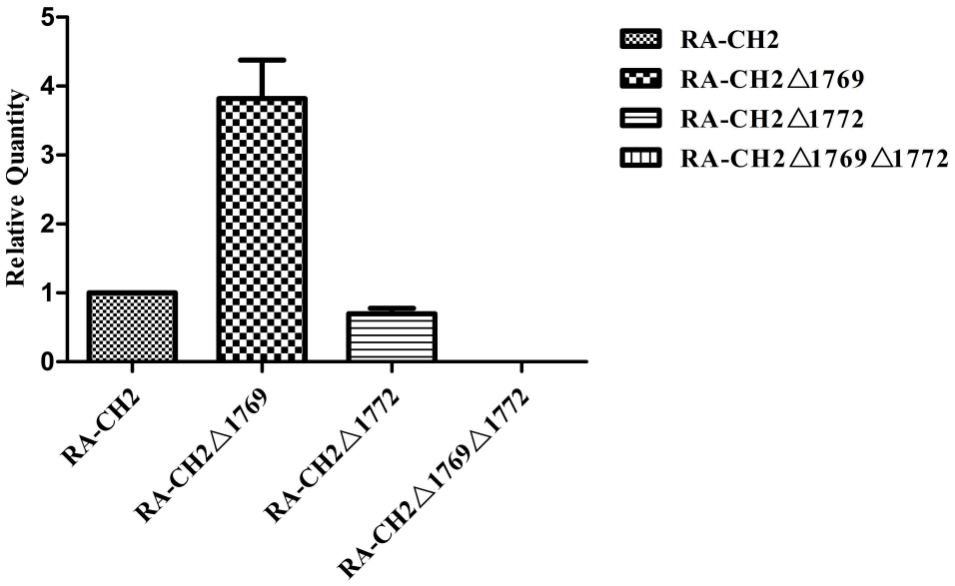
**Real-time RT-PCR analysis of ***cat*** gene expression in the RA-CH2, RA-CH2Δ1769, RA-CH2Δ1772, and RA-CH2Δ1769Δ1772 strains**. The changes of mRNAs were expressed as fold expression and calculated using the comparative CT (2^−ΔΔCT^) method. The error bars represent the standard deviation of three independent experiments.

According to the study described above, the minimum inhibitory concentrations for chloramphenicol between RA-CH2Δ1769 and RA-CH2Δ1772 are not same (Table 3). To explore whether the transcription of *cat* gene is affected by single deletion strain, RT-PCR analysis was performed. The result revealed that G148-1772 was upregulated 3.82-fold in the RA-CH2Δ1769 mutant (Figure 1). However, the mRNA level of G148-1769 in RA-CH2Δ1772 did not increased significantly. This information could explain why the resistance level of RA-CH2Δ1769 is greater than RA-CH2Δ1772 and wild-type strain. The result showed that the *cat* genes do mediate the production of chloramphenicol resistance and the relationship of the two *cat* copies is complementary and cooperative in *R. anatipestifer* CH-2.

### The transcription of *cat* gene was induced by chloramphenicol

**Figure 2:**
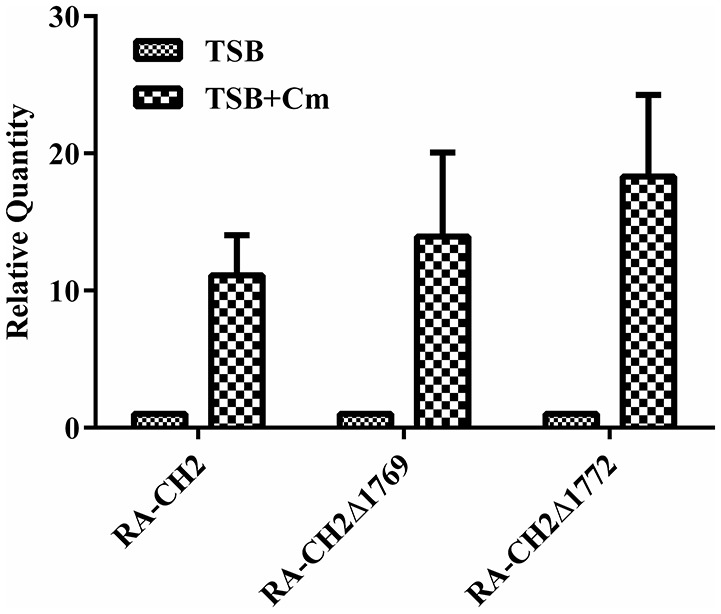
**mRNA level of ***cat*** genes in chloramphenicol-induced RA-CH2 and mutant strains RA-CH2Δ1769, RA-CH2Δ1772**. The changes of mRNAs were expressed as fold expression and calculated using the comparative CT (2^−ΔΔCT^) method. The error bars represent the standard deviation of three independent experiments.

In order to study the cellular strategies used by *R. anatipestifer* CH-2 and mutant strains in the presence of chloramphenicol, we decided to analyze transcriptional changes of *cat* gene in these strains growing in the presence of 1 μg/ml of this antibiotic. Chloramphenicol treatment had no bactericidal effect when sub-inhibitory concentration of chloramphenicol were applied (data not shown). We found that the mRNA level of *cat* genes was increased 11-fold, 13.94- and 18.31-fold in wild strain and mutant strains RA-CH2Δ1769, RA-CH2Δ1772, respectively (Figure 2). These results suggested that *cat* genes were regulated by chloramphenicol.

### Catalytic activity of the CAT and CAT^H79A^ proteins

**Figure 3:**
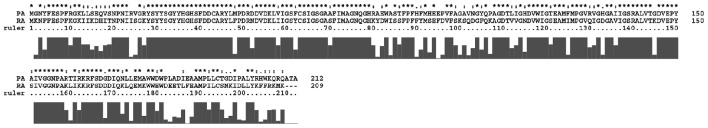
**Sequence alignments of CAT from ***R. anatipestifer*** (RA) and ***P. aeruginosa*** (PA) using CLUSTAL X**.

In a previous study involving *Pseudomonas aeruginosa*, His79 served as a major catalytic residue (Beaman et al., 1998). The two amino acid sequences of *cat* from *P. aeruginosa* and *R. anatipestifer*, respectively, showed 86.95% identity (Figure 3). To examine the main catalytic site of CAT from *R. anatipestifer* CH-2, the *cat* gene and the *cat*^*H*79*A*^ gene were expressed in *E. coli* cells. The gene products, which contained *C*-terminal His_6_-tag, were purified by Ni-agarose affinity chromatography, yielding a distinct protein band in the SDS-PAGE gel, with an approximately molecular weight of ~25 kDa. The catalytic activities of CAT and CAT^H79A^ were analyzed at 37°C. The detailed information of the reaction mixtures is described in the Materials and Methods. The specific activities of CAT and CAT^H79A^ were 8.33 ± 0.38 and 0 U·mg^−1^, respectively (Table 4). Meanwhile, the MIC of ATCC 11845 harbored *cat*^*H*79*A*^ was significantly lower than that of ATCC 11845 carried the *cat* gene (Table 3). Thus, the H79A substitution had a significant effect on CAT activity.

**Table 4:** **The CAT and CAT^**H79A**^ activity**.

**Protein**	**Specific activity (U/mg)**			
	**I**	**II**	**III**	**Avg**
CAT	8.75	8.25	8.0	8.33 ± 0.38
CAT^H79A^	0	0	0	0

## Discussion

CATs inactivate chloramphenicol via acetylation, which is the most prevalent mechanism of resistance to chloramphenicol in bacteria (Shaw, 1983; Murray and Shaw, 1997; Schwarz et al., 2004). CATs have been described in both gram-positive and gram-negative bacteria. There are two defined types of CATs that distinctly differ in their structure: The classical CATs, which are referred to as type A CATs, and the novel CATs, which are also known as type B CATs (Schwarz et al., 2004). There are at least 16 distinct groups of *catA* genes (A1–A16) and at least 5 different groups of type B *cat* genes (B1–B5) (Schwarz et al., 2004). Types A and B CATs are both capable of acetylating the hydroxyl group at C_3_ of chloramphenicol.

In our case, there was two copies of the *cat* gene in *R. anatipestifer* CH-2. Not surprisingly, the phenomenon of having 2 copies of the *cat* gene was found in other bacteria, for example *Clostridium sporogenes* (CP009225) (Zhang et al., 2015), *Chryseobacterium* sp. (AP014624) (Morohoshi et al., 2014), *M. odoratimimus* (CP013690) (Hu et al., 2016), and *Aliivibrio wodanis* (LN554847). Mutant strains were constructed. Only both two copies of the *cat* mutant strain showed a significant reduction in resistance to chloramphenicol, but not for the single *cat* gene deletion strains. ATCC 11845 is a *R. anatipestifer* strain that was isolated from ducklings in 1932, and genome analysis indicated that it does not harbor the *cat* gene and is sensitive to chloramphenicol. Complementation ATCC 11845 with the *cat* gene from *R. anatipestifer* CH-2 restored the level of chloramphenicol resistance. These results showed that the *cat* genes do mediate the production of chloramphenicol resistance and the relationship of the two *cat* copies is complementary and cooperative in *R. anatipestifer*.

To explore the function and the active site of the *cat* gene in *R. anatipestifer*, CAT and CAT^H79A^ were expressed and purified. Enzymatic activity analysis of CAT and CAT^H79A^ produced by *in vitro* site-directed mutagenesis indicated that CAT^H79A^ had no catalytic activity, thereby suggesting that His79 is the main catalytic residue of CAT. In addition, the present study further demonstrated that the *cat* gene is involved in chloramphenicol resistance, thus supporting our hypothesis that the *cat* genes are chloramphenicol resistance determinant factors in *R. anatipestifer*.

**Figure 4:**
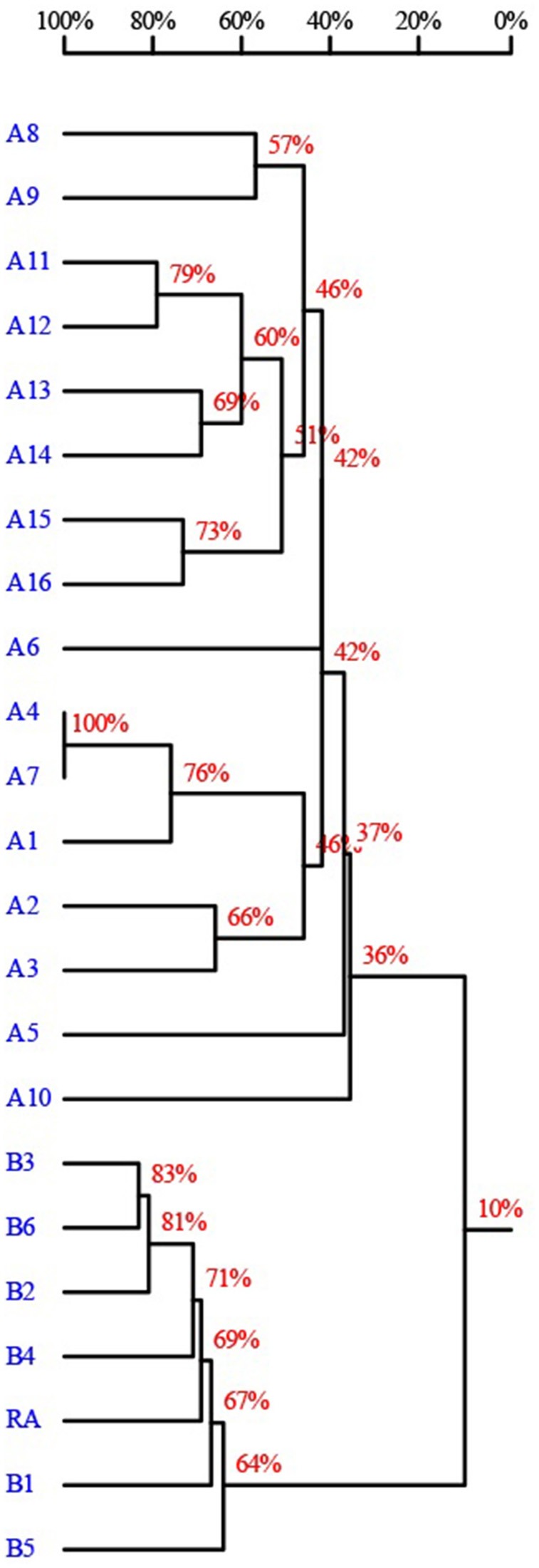
**Homology analysis of types A and B CATs based on amino acid identity using DNAMAN 8.0 (Lynnon-Biosoft, Ontario, Canada). The sequence information of types A and B CATs are listed in the Supplementary Table 1**.

Type B CATs can be further classified into at least five groups. We constructed a homology tree of types A and B CATs (Figure 4) based on their reported amino acid sequence (van Hoek et al., 2011; Roberts et al., 2012). Types A and B CATs showed 10% similarity. We also determined that the *R. anatipestifer* CAT forms a separate branch from the type B CATs. In addition, types A4 and A7 CATs were observed to be 100% similarity. Thus, the classification of CATs should be revisited. Two types of genes that encode CATs could be based on their structure, namely, types A and B, by using the criterion of ≥80% amino acid identity to define a subgroup (Roberts and Schwarz, 2009). The sequence information of types A and B CATs is listed in the Supplementary Table 1. A total of 15 distinct groups were identified, A1–A15 for type A CATs and five different groups for type B CATs, B1–B5. Types A4 and A7 share 100% identity and belong to a subclass that we designated as A4. Groups A8–A16 were renamed as A7–A15. Groups B2, B3, and B6 showed >80% homology. These three categories are classified as a subclass, namely, B2. The CAT of *R. anatipestifer* was designated as B3. The rest of the type B classifications remained the same.

It was reported that the *cat* genes identified in gram-positive bacteria *Bacillus* spp. and *Straphylococcus* were inducibly expressed by chloramphenicol (Mongkolsuk et al., 1984; Bruckner and Matzura, 1985; Duvall et al., 1985). To verify whether the *cat* genes were induced by chloramphenicol in *R. anatipestifer* CH-2, RT-PCR was performed to determine the *cat* transcript level of the wild-type strain and mutant strains in the presence or absence of chloramphenicol at a concentration of 1 μg/ml. The results exhibited that the level of transcription of the *cat* gene increased in the presence of chloramphenicol. However, the inducing mechanism is not understood at this time in *R. anatipestifer*.

It has been demonstrated that *catA86* and *catA112* were regulated by a mechanism known as translation attenuation in the previous studies (Lovett, 1996). Later, translational attenuation has been proposed as the regulatory mechanism for the chloramphenicol-inducible *catB1* gene of *Agrobacterium tumefaciens* (Rogers et al., 2002). Sequence analysis found that CAT from *R. anatipestifer* shared 65% identity with that of *A. tumefaciens*. It is unclear whether they have the same inducing mechanism. Further studies determining the regulatory mechanism underlying the *cat* gene in *R. anatipestifer* are warranted.

## Author contributions

DZ and AC conceived and designed the project; LH and HY constructed the *cat* deletion mutant of *R. anatipestifer* and detected resistance; ML and XZ detected the mRNA levels of the *cat* gene by RT-PCR; LH and HY constructed ATCC 11845 *cat* and *cat*^*H*79*A*^ complementary strains. LH, RJ, and SC performed expression and purification of CAT and CAT^H79A^ His-tagged proteins; LH, QY, and YW performed CAT activity assay; MW, KS, and XC detected the *cat* gene in *R. anatipestifer* isolates; LH and DZ drafted and revised the manuscript. All authors have read and approved the final version manuscript.

## Funding

This work was supported by the National Natural Science Foundation of China under Grant No. 31372468; National Science and Technology Support Program under Grant No. 2015BAD12B05; China Agricultural Research System under Grant No. CARS-43-8; Youth Science and Technology Innovation Research Team of Sichuan Province for Waterfowl Diseases Prevention and Control under Grant No. 2013TD0015; Integration and Demonstration of Key Technologies for Duck Industrial in Sichuan Province under Grant No. 2014NZ0030.

### Conflict of interest statement

The authors declare that the research was conducted in the absence of any commercial or financial relationships that could be construed as a potential conflict of interest.
