# Supplementary material for: Type B Chloramphenicol Acetyltransferases Are Responsible for Chloramphenicol Resistance in Riemerella anatipestifer, China
Source: Front Microbiol. 2017 Mar 1;8:297. doi: 10.3389/fmicb.2017.00297 (PMC5331189; doi:10.3389/fmicb.2017.00297)
Supplement: Supplementary file 1 [file Table1.DOCX]

Supplementary Table 1 The sequence of type A CATs and type B CATs

| Group | Gene | Genera | Accession number | Protein ID | Located |
| --- | --- | --- | --- | --- | --- |
| TypeA-1 | *cat* | *Escherichia coli* | V00622 | CAA23899 | Tn9 |
| TypeA-2 | *II-CAT* | *Escherichia coli* | X53796 | CAA37805 | Chromosome |
| TypeA-3 | *catIII* | *Shigella flexineri* | X07848 | CAA30695 | pR387 |
| TypeA-4 | *cat* | *Proteus mirabilis* | M11587 | AAA25655 | Chromosome |
| TypeA-5 | *cat* | *Streptomyces acrimycini* | P20074 | JQ0375 | Chromosome |
| TypeA-6 | *cat86* | *Bacillus subtilis* | K00544 | AAA22289 | Chromosome |
| TypeA-7 | *cat* | *Staphylococcus aureus* | X02529 | CAA26367 | pC221 |
| TypeA-8 | *cat* | *Staphylococcus aureus* | AY355285 | AAQ55242 | pC223 |
| TypeA-9 | *cat* | *Staphylococcus aureus* | NC_002013 | NP_040437 | pC194 |
| TypeA-10 | *cat* | *Bacillus clausii* | AY238971 | AAQ63644 | Chromosome |
| TypeA-11 | *catP* | *Clostridium perfringens* | U15027 | AAB51421 | Tn4451 |
| TypeA-12 | *catS* | *Streptococcus pyogenes* | X74948 | CAA52904 | Chromosome |
| TypeA-13 | *cat* | *Campylobacter coli* | M35190 | AAA23018 | pC-589 |
| TypeA-14 | *cat* | *Listonella anguillarum* | S48276 | AAB23649 | pJA7324 |
| TypeA-15 | *catB* | *Clostridium butyricum* | M93113 | AAA73865 | Chromosome |
| TypeA-16 | *catQ* | *Clostridium perfringens* | M55620 | AAA23215 | Chromosome |
| TypeB-1 | *cat* | *Agrobacterium fabrum* | M58472 | AAA22081 | Chromosome |
| TypeB-2 | *catB2* | *Escherichia coli* | AF047479 | AAC14737 | pNR79 |
| TypeB-3 | *catB3* | *Salmonella typhimurium* | AJ009818 | CAA08841 | pBWH301 |
| TypeB-4 | *catB7* | *Pseudomonas aeruginosa* | AF036933 | AAD02068 | Chromosome |
| TypeB-5 | *catB9* | *Vibrio cholerae* | AF462019 | AAL68645 | Chromosome |
| TypeB-6 | *catB10* | *Pseudomonas aeruginosa* | FJ495083 | ACL13298 | TS-832035 |
